# Lung CT stabilization with high-frequency non-invasive ventilation (HF-NIV) and breath-hold (BH) in lung nodule assessment by PET/CT

**DOI:** 10.1186/s41824-023-00175-4

**Published:** 2023-09-04

**Authors:** Mario Jreige, Emeline Darçot, Alban Lovis, Julien Simons, Marie Nicod-Lalonde, Niklaus Schaefer, Flore Buela, Olivier Long, Catherine Beigelman-Aubry, John O. Prior

**Affiliations:** 1 Department of Nuclear Medicine and Molecular Imaging, Lausanne University Hospital, Rue du Bugnon 46, 1011 Lausanne, Switzerland; 2 Department of Diagnostic and Interventional Radiology, Lausanne University Hospital, Lausanne, Switzerland; 3 Department of Pulmonology, Lausanne University Hospital, Lausanne, Switzerland; 4 Faculty of Biology and Medicine, University of Lausanne, Lausanne, Switzerland; 5 Department of Physiotherapy, Lausanne University Hospital, Lausanne, Switzerland

**Keywords:** Pulmonary nodules, PET/CT, FDG, HF-NIV

## Abstract

**Purpose:**

To evaluate the effect of lung stabilization using high-frequency non-invasive ventilation (HF-NIV) and breath-hold (BH) techniques on lung nodule detection and texture assessment in PET/CT compared to a free-breathing (FB) standard lung CT acquisition in PET/CT.

**Materials and methods:**

Six patients aged 65 ± 7 years, addressed for initial assessment of at least one suspicious lung nodule with ^18^F-FDG PET/CT, underwent three consecutive lung PET/CT acquisitions with FB, HF-NIV and BH. Lung nodules were assessed on all three CT acquisitions of the PET/CT and characterized for any size, volume and solid/sub-solid nature.

**Results:**

BH detected a significantly higher number of nodules (*n* = 422) compared to HF-NIV (*n* = 368) and FB (*n* = 191) (*p* < 0.001). The mean nodule size (mm) was 2.4 ± 2.1, 2.6 ± 1.9 and 3.2 ± 2.4 in BH, HF-NIV and FB, respectively, for long axis and 1.5 ± 1.3, 1.6 ± 1.2 and 2.1 ± 1.7 in BH, HF-NIV and FB, respectively, for short axis. Long- and short-axis diameters were significantly different between BH and FB (*p* < 0.001) and between HF-NIV and FB (*p* < 0.001 and *p* = 0.008), but not between BH and HF-NIV. A trend for higher volume was shown in FB compared to BH (*p* = 0.055) and HF-NIV (*p* = 0.068) without significant difference between BH and HF-NIV (*p* = 1). We found a significant difference in detectability of sub-solid nodules between the three acquisitions, with BH showing a higher number of sub-solid nodules (*n* = 128) compared to HF-NIV (*n* = 72) and FB (*n* = 44) (*p* = 0.002).

**Conclusion:**

We observed a higher detection rate of pulmonary nodules on CT under BH or HF-NIV conditions applied to PET/CT than with FB. BH and HF-NIV demonstrated comparable texture assessment and performed better than FB in assessing size and volume. BH showed a better performance for detecting sub-solid nodules compared to HF-NIV and FB. The addition of BH or HF-NIV to PET/CT can help improve the detection and texture characterization of lung nodules by CT, therefore improving the accuracy of oncological lung disease assessment. The ease of use of BH and its added value should prompt its use in routine practice.

## Introduction

Fluorine-18-fluorodeoxyglucose (^18^F-FDG) PET/CT is recommended for the evaluation of patients with stage I to IV of non-small cell lung cancer (NSCLC) according to the guidelines of the National Comprehensive Cancer Network (NCCN), the Society of Nuclear Medicine and Molecular Imaging, the American College of Chest Physicians and the American College of Radiology Appropriateness Criteria (Ettinger et al. 2017; Silvestri et al. 2013; Ravenel et al. 2014). ^18^F-FDG PET/CT also plays an important role in the evaluation of patients with small cell lung cancer (SCLC) and in the detection of secondary lung nodules (Martucci et al. 2020; Bamba et al. 2011). The performance of PET/CT in detecting pulmonary nodules is known to be size-dependent (Tang et al. 2019). Digital PET/CT technological improvement allowed the detection of smaller nodules, but the lowest detectability threshold of PET/CT remains worse than that of high-resolution CT scan at full inspiration (Groheux et al. 2016; Hagi et al. 2018; Meyer et al. 2020). The aim of this study is to evaluate the effect of lung stabilization at full inspiration using high-frequency non-invasive ventilation (HF-NIV) and breath-hold (BH) techniques on lung nodule detection and texture characterization using the CT part of PET/CT compared to a free-breathing (FB) standard CT acquisition for lung analysis.

## Materials and methods

### Study design

Permission from the State of Vaud ethic committee (CER-VD, 2018-00438) was obtained for this prospective study. All volunteers provided written informed consent prior any study-associated procedure.

### Patients’ cohort

Only two inclusion criteria were defined: age 18 years or older and presence of at least 1 non-calcified suspicious pulmonary nodule ≥ 6 mm, whatever its texture (solid, sub-solid), incidentally discovered or diagnosed in a follow-up setting. The exclusion criteria are summarized in Table 1.

**Table 1:** Exclusion criteria

Previous or current disorders that might interfere with performance or safety of study procedures
Age < 18 years
Pregnant or breastfeeding women
Any contraindication to MRI (pacemakers, neurostimulators, some implantable devices, some metallic implants, claustrophobia)^a^
Adults with mental incapacities
Inability to follow the procedures of the study e.g. due to language problems, psychological disorders, dementia, etc. of the participant
COPD or asthma with severe obstruction: severe obstructive patients (FEV1 < 50% of predicted value), Hypoxemia (SaO_2_ < 94% AA), history or physical signs of right heart failure
History or physical signs of right or left cardiac failure
History or physical signs of pulmonary hypertension
History or physical signs of active coronary artery disease
Pulmonary graft
Immunocompromised patients
Known or suspected non-compliance with appointments, alcoholism, drug addiction or alike
Enrolment of the investigator, his/her family members, employees and other dependent persons

Respiratory and cardiac conditions listed in this table are defined according to the use of the HF-NIV

*COPD* chronic obstructive pulmonary disease, *FEV* forced expiratory volume, *SaO*_*2*_ oxygen saturation rate

^a^These exclusion criteria were applicable on both MRI and PET/CT sub-studies sharing the same patient population

**Fig. 1:**
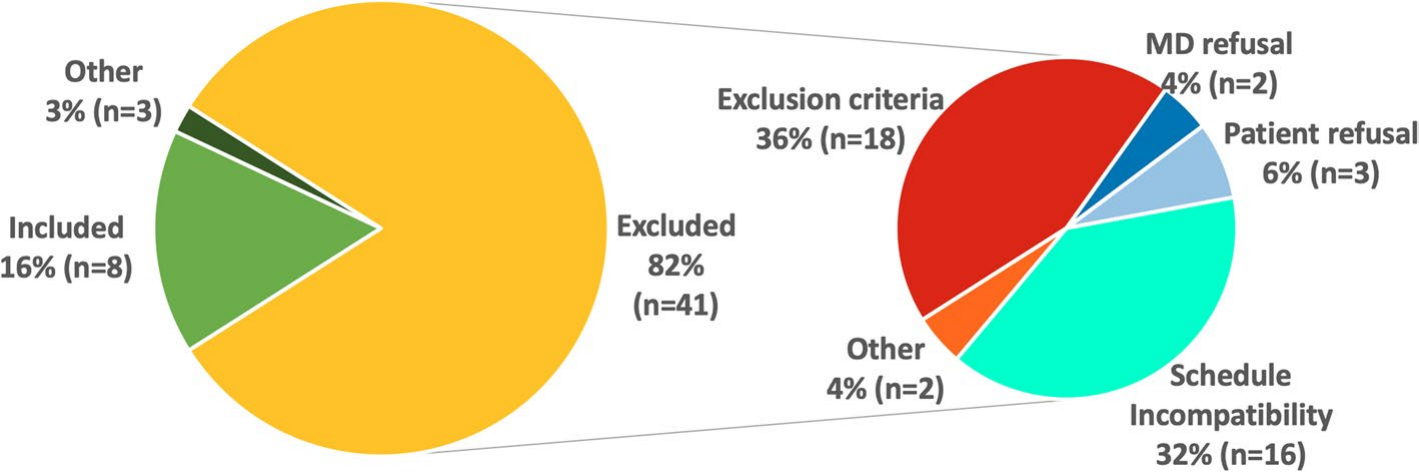
Details of the patient inclusion and exclusion during the screening

Fifty-two patients satisfying the inclusion criteria were identified, of whom forty-four (85%) were not eligible for various causes (Fig. 1). Of the 8 patients included, 7 completed the study (age 66 ± 9 years, weight 73.4 ± 10.2 kg, 3 women) and one was unable to undergo PET/CT exam with HF-NIV.

### HF-NIV technique

HF-NIV was achieved with a Monsoon III ventilator (Acutronic Medical Systems, Hirzel, Switzerland) and a non-invasive patient interface (Phasitron, Percussionnaire, Idaho, USA) (Beigelman-Aubry et al. 2017), according to a set-up described in Delacoste et al. (2019). Oxygen delivery was set to 100% on the monitor. In practice, given the inlet of ambient air through the inspiratory port of the Phasitron®, the fraction of inspired oxygen (FiO_2_) was ~ 50% (Darcot et al. 2020).

To assess the tolerance to the HF-NIV and to ensure a respiratory stabilization period ≥ 6 min at a respiratory rate of 250/min in agreement with Ogna et al. (2017), each patient was trained by a physiotherapist before the PET/CT examination during a screening session. In addition, the following physiological parameters were monitored by a pulmonologist with a Digital Monitoring System (SenTec, Therwil, Switzerland): continuous transcutaneous capnography (TcCO_2_), oxygen saturation (SpO_2_) and cardiac frequency. Arterial pressure was also monitored.

### ^18^F-FDG–PET/CT acquisitions

Patients underwent ^18^F-FDG–PET/CT on a Vision PET/CT system (Siemens Healthineers) 60–78 min after a planned intravenous injection of 2 MBq/kg of ^18^F-FDG. All patients fasted for at least 6 h and had blood glucose levels lower than 140 mg/dl before administration of ^18^F-FDG.

A FB low-dose helical CT (tube voltage automodulated by CARE kV (Siemens Healthcare) system with a reference voltage of 100 kV, tube current automodulated by CARE Dose 4D (Siemens Healthcare) with a reference current value of 100 mA, pitch 1.2, 0.5 s/rotation, 2 mm slice thickness) was first performed for anatomical correlation and attenuation correction.

A HF-NIV lung PET/CT was subsequently acquired with a CT acquisition protocol according to the following parameters: tube voltage automodulated by CARE kV (Siemens Healthcare) system with a reference voltage of 80 kV, tube current automodulated by CARE Dose 4D (Siemens Healthcare) with a reference current value of 20 mA, pitch 1.2, 0.5 s/rotation, 2 mm slice thickness.

Lastly, a lung PET/CT acquisition was acquired during a single BH with an average duration of 20 s for PET and 10 s for CT scan using the following parameters: tube voltage automodulated by CARE kV (Siemens Healthcare) system with a reference voltage of 80 kV, tube current automodulated by CARE Dose 4D (Siemens Healthcare) with a reference current value of 20 mA, pitch 1.2, 0.5 s/rotation, 2 mm slice thickness.

The different ventilation techniques were acquired for the CT and PET parts during the same procedure under full inspiration apnea for the HF-NIV and BH techniques, for CT and PET scans were acquired without the requirement for apnea.

For the three successive acquisition protocols, all raw data were reconstructed by iterative reconstruction algorithms using soft and lung kernel with 1 mm slice thickness.

### Nodule detection and evaluation

CT analysis of nodules from the three different acquisitions (FB, HF-NIV and BH) was performed by two radiologists with 25 and 5 years of experience in thoracic imaging by consensus reading on anonymized and randomly sorted images. Lung nodules were assessed on all three CT acquisitions and recorded according to the following visual score: absent = 1; probably absent = 2; uncertain = 3; probably present = 4; present = 5. They were characterized for two-dimensional (2D) size using long (*l*_axis_) and short (*s*_axis_) axis (mm), as well as volume (mm^3^) and texture (solid/subsolid). The *l*_axis,_ *s*_axis_ and volume measurements were compared between the three CT acquisitions.

Nodule volume was measured with the lesion management software (Carestream, Rochester, New York, USA) using soft kernel reconstructions. The volumetric measurements performed on soft kernel images by the most experienced reader were considered the reference value. Semi-automatic contouring was used by default and manual drawing was also applied when necessary.

### Statistical analyses

All statistical analyses were performed using STATA version 14.0 (STATA Corp., College Station, TX, USA). Continuous variables are presented as mean ± standard deviation or median (interquartile range [IQR]). Ordinal data are reported as number or percentage and compared by means of Fisher’s exact or chi-squared test as appropriate. All collected variables derived from the texture and volumetric analysis of different CT acquisitions were then compared using the one-way analysis-of-variance (ANOVA) model. The analysis was applied to subgroups based on nodule size, using a cutoff of 3 mm for *l*_axis_ to investigate the nodule measurements dependency on *l*_axis_. *p* values < *0.05* were considered statistically significant.

## Results

### Patient’s cohort

Of the 8/52 (15%) included patients, all but one (age 66 ± 9 years, weight 73.4 ± 10.2 kg, 3 women) successfully completed the PET/CT scan under-HF-NIV stabilization period: 4.45 min, range [3–6.5] without any adverse events. One patient was unable to undergo PET/CT with HF-NIV due to inability to follow the instructions.

### Nodule detection

**Fig. 2:**
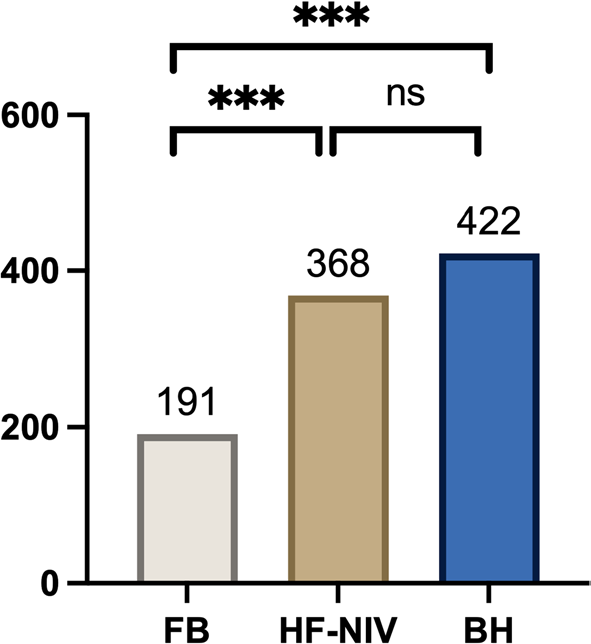
Number of detected lung nodules. Legend: BH: Breath Hold, HF-NIV: High Frequency Non-Invasive Ventilation, FB: Free Breathing, ns: not significant, ***p < 0.001 versus BH

BH detected a significantly higher number of nodules (*n* = 422) compared to HF-NIV (*n* = 368 or 87.2% of the total BH nodules) and FB (*n* = 191, 45.3%) (*p* < 0.001) (Fig. 2).

A significant difference in nodule visual score between BH, HF-NIV and FB was also noticed (*p* < 0.001). The total number of nodules detected by BH with an assigned score above 4 was 307, significantly higher than those detected by HF-NIV (*n* = 292) and FB (*n* = 173) (*p* < 0.001) (Table 2).

**Table 2:** Characteristics of lung nodules

	FB	HF-NIV	BH	*p* value
Number of nodules	191***	368***	422	< 0.001
*l*_axis_ (mm)	3.2 ± 2.4***	2.6 ± 1.9	2.4 ± 2.1	< 0.001
*s*_axis_ (mm)	2.1 ± 1.7***	1.6 ± 1.2	1.5 ± 3	< 0.001
Nodule volume (mm^3^)	49.8 ± 202.1^†^	23 ± 112	22.6 ± 105.1	0.040
Solid/sub-solid pattern	147 (76.9%)/44 (23%)**	296 (80.4%)/72 (19.6%)**	294 (69.7%)/128 (30.3%)	0.002

*BH* breath hold, *HF-NIV* high frequency non-invasive ventilation, *FB* free breathing

***p < 0.001, **p < 0.01, ^†^*p* < 0.1 versus BH

### ***s***_axis_ and ***l***_axis_ analysis

The mean measured nodule size (mm) was 2.4 ± 2.1, 2.6 ± 1.9 and 3.2 ± 2.4 in BH, HF-NIV and FB, respectively, for long axis and 1.5 ± 1.3, 1.6 ± 1.2 and 2.1 ± 1.7 in BH, HF-NIV and FB, respectively, for short axis. We found a statistically significant difference between BH and FB (*p* < 0.001 and *p* < 0.001) and between HF-NIV and FB (*p* < 0.001 and p = 0.008) for *s*_axis_ and *l*_axis_, but no significant difference between BH and HF-NIV (*p* = 1 and *p* = 0.409). For nodules with *l*_axis_ less than 3 mm, there was a significant difference in *l*_axis_ measurement between BH (1.4 ± 0.5), HF-NIV (1.7 ± 0.5) and FB (1.8 ± 0.4) (*p* < 0.001).

### Volumetric analysis

**Fig. 3:**
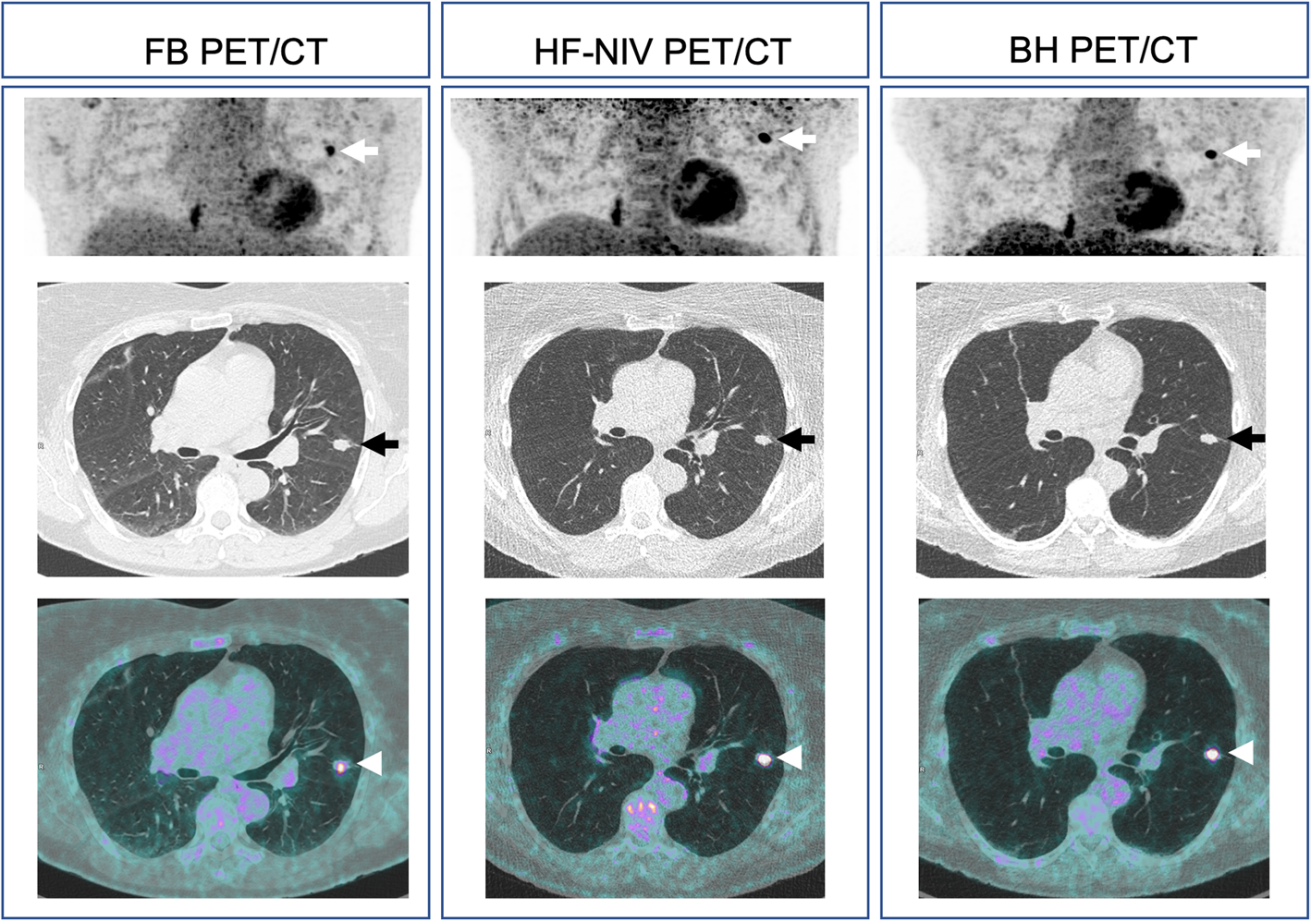
Example. A 60-year-old female patient with a history of splenic marginal zone lymphoma underwent PET/CT for characterization of a left lower lobe pulmonary nodule, shown on PET maximal intensity projection (white arrows), CT images with lung kernel (black arrows) and fusion images (arrow heads). The measured nodule long axis (mm), short axis (mm) and volume (mm^3^) were 17, 9 and 1003 on FB, 16, 9 and 802 on HF-NIV and 15, 7 and 793 on BH

The mean nodule volume (mm^3^) was 22.6 ± 105.1 in BH, 23 ± 112 in HF-NIV and 49.8 ± 202.1 in FB. A trend for higher measured volume was shown in FB compared to BH (*p* = 0.055) and HF-NIV (*p* = 0.068) without significant difference between BH and HF-NIV (*p* = 1) (Fig. 3).

For nodules measuring less than 3 mm in *l*_axis_, there was a significant difference in measured nodule volume between BH (5.4 ± 2.5) and HF-NIV (4.8 ± 2.3) (*p* = 0.004), BH and FB (6.4 ± 3) (*p* = 0.010) and between HF-NIV and BH (*p* < 0.001).

For nodules measuring more than 3 mm in *l*_axis_, there was a significant difference in nodule volume between BH (57.3 ± 177.8) and HF-NIV (52.7 ± 177.9) (*p* = 1), BH and FB (86.2 ± 269.1) (*p* = 0.841) and between HF-NIV and BH (*p* = 0.636).

### Solid/sub-solid assessment

We found a significant difference in detectability of sub-solid nodules between the three acquisitions with BH showing a higher number of detected sub-solid nodules (*n* = 128) compared to HF-NIV (*n* = 72) and FB (*n* = 44) (*p* = 0.002).

## Discussion

In this study, we showed that application of lung stabilization techniques—more specifically BH and HF-NIV—during PET/CT resulted in the identification of a higher number of nodules than the FB CT acquisition traditionally used in PET/CT. We found a statistically significant difference in 2D lung measurements on CT acquired under lung stabilization compared to FB, with lower values measured with BH. However, no significant differences were noted between BH and HF-NIV for 2D measurements. A trend for higher volume measurement was shown in FB compared to BH and HF-NIV without significant difference between BH and HF-NIV. The detectability of sub-solid nodules was significantly higher in BH and HF-NIV than in FB.

Computed tomography plays a crucial role in lung nodule assessment in particular in PET/CT imaging. It allows adding important information about size, location, as well as solid/subsolid texture, margins and internal content of nodules (MacMahon et al. 2017). These findings, when added to the PET tracer uptake data, can help to better characterize the lung nodule nature. In conjunction with the recent improvement in PET/CT spatial resolution (Groheux et al. 2016; Hagi et al. 2018; Meyer et al. 2020), the addition of BH acquisition to routine techniques allows to acquire a lung-dedicated PET/CT during a maximum of 30 s of breath-holding at full inspiration. This technique has the advantage of improving the tracer uptake quantification while obtaining a stabilized CT acquisition at full inspiration allowing an optimal lung analysis (Bärwolf et al. 2017). In concordance with previous studies targeting oncological applications, our study has shown a clear benefit of BH in increasing the number of nodules detected as well as the accuracy of 2D and 3D measurements and nodule texture assessment in patients addressed for lung nodules evaluation, overcoming the limited image quality in FB due to respiratory motion blurring effect (Meirelles et al. 2007; Balamoutoff et al. 2018).

Differentiation of solid nodules from part-solid ones was difficult to make due to the blurring of the image acquired in FB. Furthermore, despite a rigorous drawing, the volume estimation was often difficult to assess due to the noise in the image and most of the nodules in FB required a manual extraction after failure of the auto-segmentation.

Other applications of ^18^F-FDG PET/CT in inflammatory diseases such as lung sarcoidosis could benefit from the improved detectability of lung nodules, for both initial diagnosis and response assessment to therapy. Some infectious disorders such as fungal disease could also benefit from the technique. In addition, the aim of this study was to address the early detection of lung metastases especially tiny ones in young patients suffering from cancer such as sarcoma and therefore potentially improve the overall management. In this way, nodules of any size were evaluated.

Increasing the detectability and improving the morphologic characterization of lung nodules may also positively affect the quantitative analysis of CT radiomics features derived from PET/CT. In fact, CT radiomics features derived from diagnostic high-resolution thoracic CT showed promising results in classification of lung nodules, determination of histology and genomic and treatment outcomes predictions (Ayachy et al. 2021).

As for volumetric nodule assessment, it is considered a major component of the evaluation of benignancy or malignancy of nodules of intermediate nature by the measurement of their volume doubling time (Devaraj et al. 2017). Nodule volumetric assessment depends on numerous factors including the value of the whole lung volume (Goo et al. 2006). In this way, in order to accurately evaluate the volume doubling time between the PET/CT and the previous diagnostic thoracic CT examinations, the lung volume has to be similar to that of the diagnostic thoracic CT performed at full inspiration. This goal can be achieved with the integration of BH or HF-NIF within the current protocol.

To our knowledge, this is the first study investigating lung nodule detection and characterization on the CT component of PET/CT under HF-NIV in comparison to FB acquisition. In a previously published study from our institution, a first-time clinical evaluation of the reduction of respiratory motion during PET/CT by pulsatile-flow ventilation showed a reduction in thoracic organ motion and an increase in lesion standard uptake values (SUV) under HF-NIV (Prior et al. 2016). Our study confirms the interest of such a technique by also improving nodule detectability and delineation previously mentioned. Interestingly, HF-NIV performance was comparable to that of BH at full inspiration for 2D and 3D volume measurements. These results suggest that HF-NIV can be used as an alternative to BH for accurate lung nodule assessment in sick patients who cannot maintain an apnea during up to 30 s. On the other hand, BH is an easier technique that does not require a physiotherapist expertise nor any additional equipment.

Based on our current evaluation, the image quality obtained with both BH and HF-NIV was of diagnostic value. Therefore, these techniques could theoretically replace dedicated lung CT. However, in this study, we did not compare the image quality between dedicated lung CT and the CT part of the BH and HF-NIV procedures, which should be studied and confirmed by a larger cohort.

We want to address some limitations of our study. The main limitation is the relative low number of patients included. However, the ability of all patients to maintain a sufficient BH and to cooperate adequately with the HF-NIV technique, as well as the high number of total nodules detected allowed the performance of a nodule-based analysis. Nevertheless, a larger patient group is still required to confirm our findings. Next, studying small nodules that were the commonest in our study is debatable. Nevertheless, any earlier detection of a tumoral lesion may be considered as a potential progress in the management of patients care. Indeed, if a volume lower than 50 mm^3^ is not considered to be actionable in the context of an incidental discovery or during a screening, any discovery or increase of a nodule whatever its size in a cancer patient may represent an indicator of progression. Another limitation is that we measured the absolute 2D value of any nodule with a 0.1 mm difference which is far below the human mistake of measurement with a caliper (Revel et al. 2004), and we didn’t consider the 0.5 mm lower or higher nominal value as recommended in the literature (Bankier et al. 2017). Nevertheless, we wanted to be as precise as possible during our concurrent reading, this in order to ensure a strict comparison between acquisitions. To add, the availability of the equipment used in this study may not be currently widely available and this could be a limitation in the application of the proposed technique. Finally, in addition to nodule detectability and characterization, the quantitative analysis of tracer uptake that could benefit from lung stabilization, especially for small nodules, should also be evaluated. This was out of the scope of the present study and should be further addressed to fully evaluate the usefulness of these techniques on SUV measurements.

## Conclusion

A higher detection rate of pulmonary nodules may be assessed on lung CT acquired under BH or HF-NIV conditions applied to PET/CT than in FB. BH and HF-NIV demonstrated comparable pulmonary nodule morphologic characterization and performed better than FB in assessing nodules size and volume. BH showed a better performance regarding the detection of sub-solid nodules compared to HF-NIV and FB. Therefore, the addition of BH or HF-NIV to PET/CT, allowing lung stabilization at full inspiration, could improve the accuracy of oncological lung disease assessment. The easier feasibility of the former without requiring neither any expertise in ventilation technique nor any additional equipment should prompt its use in routine practice in a near future.

## Data Availability

The data sets used and analysed during the current study are available from the corresponding author on reasonable request.

## Abbreviations

HF-NIV: High-frequency non-invasive ventilation
BH: Breath-hold
FB: Free-breathing
